# Multiplexed methylation profiles of tumor suppressor genes and clinical outcome in oligodendroglial tumors

**DOI:** 10.1002/cam4.762

**Published:** 2016-07-01

**Authors:** Lu‐Ting Kuo, Hsueh‐Yi Lu, Chien‐Chang Lee, Jui‐Chang Tsai, Hong‐Shiee Lai, Ham‐Min Tseng, Meng‐Fai Kuo, Yong‐Kwang Tu

**Affiliations:** ^1^ Division of NeurosurgeryDepartment of SurgeryNational Taiwan University HospitalTaipei 100Taiwan; ^2^ Department of Industrial Engineering and ManagementNational Yunlin University of Science and TechnologyDouliuYunlin county640Taiwan; ^3^ Department of Emergency MedicineNational Taiwan University HospitalYun‐Lin branchYun‐Lin county640Taiwan; ^4^ Department of SurgeryNational Taiwan University HospitalTaipeiTaiwan

**Keywords:** ESR1, IGSF4, methylation, multiplex ligation‐dependent probe amplification assay, oligodendroglioma

## Abstract

Aberrant methylation has been associated with transcriptional inactivation of tumor‐related genes in a wide spectrum of human neoplasms. The influence of DNA methylation in oligodendroglial tumors is not fully understood. Genomic DNA was isolated from 61 oligodendroglial tumors for analysis of methylation using methylation‐specific multiplex ligation‐dependent probe amplification assay (MS‐MLPA). We correlated methylation status with clinicopathological findings and outcome. The genes found to be most frequently methylated in oligodendroglial tumors were RASSF1A (80.3%), CASP8 (70.5%), and CDKN2A (52.5%). Kaplan–Meier survival curve analysis demonstrated longer duration of progression‐free survival in patients with 19q loss, aged less than 38 years, and with a proliferative index of less than 5%. Methylation of the ESR1 promoter is significantly associated with shorter duration of overall survival and progression‐free survival, and that methylation of IGSF4 and RASSF1A is significantly associated with shorter duration of progression‐free survival. However, none of the methylation status of ESR1, IGSF4, and RASSF1A was of prognostic value for survival in a multivariate Cox model. A number of novel and interesting epigenetic alterations were identified in this study. The findings highlight the importance of methylation profiles in oligodendroglial tumors and their possible involvement in tumorigenesis.

## Introduction

Epigenetic modifications, including mainly DNA methylation and histone modification, are known to modify gene expression patterns and control different biological processes such as cell differentiation and proliferation. Aberrant methylation of CpG islands has been demonstrated to be a common event associated with transcriptional inactivation of tumor‐related genes in a wide spectrum of human neoplasms 1, 2. For example, aberrant DNA methylation in sporadic colorectal cancer has been demonstrated to be predominantly involved in the early events during malignant phenotype progression 3, 4. O^6^‐Methyl guanine methyl transferase (MGMT), a DNA repair enzyme, is hypothesized to play a role in repairing the DNA alkylation that occurs at the O^6^‐position of guanine by nitrosourea and temozolomide compounds during chemotherapy in a manner that ultimately leads to resistance to these compounds 5, 6. Recent studies found that the methylation of the promoter of the AGT gene at 10q26, which encodes the MGMT protein, leads to transcriptional inactivation of the gene, thereby increasing chemosensitivity in gliomas, especially glioblastomas. Although methylation of tumor‐related genes, such as MGMT has been detected in other types of brain tumors, including oligodendroglial tumors, meningiomas and ependymomas, the association between methylation status of these genes and progression‐free or overall survival has not been completely examined 7, 8, 9. Nevertheless, detection of gene methylation may prove essential in not only diagnosis but also therapeutic response and prognostic prediction.

Methylation‐specific multiplex ligation‐dependent probe amplification (MS‐MLPA) is a polymerase chain reaction (PCR)‐based technique that allows for identification of the methylation status of multiple genes in a single experiment 10. Based on the annealing of probes containing a recognition site for the methylation‐sensitive restriction enzyme *Hha*I, this technique has been applied in several studies of cancer 11. On digestion of the sample DNA with *Hha*I, probes designed to recognize *Hha*I sites within unmethylated regions will not generate a signal, as these sequences have been cut by *Hha*I and cannot bind to the probe. This study used this innovative technique to determine the methylation status of 24 genes in oligodendroglial tumors and correlate methylation status with clinical outcome.

## Materials and Methods

### Ethics statement

All participants provided their written consent to participate in this study, which was approved by the committee on human studies of NTUH (National Taiwan University Hospital).

### Patient population and data collection

Archival specimens of 61 oligodendroglial tumors obtained after surgery on patients, all identified as ethnically Chinese at the National Taiwan University Hospital (NTUH) between January 1994 and December 2005, were examined. The study was approved by the Institutional Review Board and informed consent was obtained from the patients. The histopathology of the tumors, of which 39 were World Health Organization (WHO) grade II oligodendrogliomas, 7 grade II oligoastrocytomas, and 15 grade III oligodendrogliomas, was reviewed by two pathologists blind to the patient data (Table 1).

**Table 1:** Patient demographic and clinicopathologic parameters

	Parameter	No. (%)
Gender	Male	34 (55.7)
	Female	27 (44.3)
Age	Mean (SD), years	37.7 (13.5)
	Range, years	11–82
Histology	Grade II oligodendroglioma	39
	Grade II oligoastrocytoma	7
	Grade III oligodendroglioma	15
	Grade III oligoastrocytoma	0
Follow‐up	Mean (SD), months	87.1 (48.1)
	Range, months	15–195

SD, standard deviation.

### DNA extraction and MS‐MLPA

After hematoxylin and eosin evaluation had been performed to ensure that a minimum of 80% of cells were tumorous, DNA was extracted from paraffin‐embedded tissues using the Genomic DNA Mini Kit (Geneaid, Taipei county, Taiwan), followed by verification of the concentration and purity of the DNA samples. MS‐MLPA was performed using the ME002 Kit (MRC‐Holland, Amsterdam, Netherlands), which simultaneously checks for methylation at one or two CpG dinucleotides of 24 proven or suspected tumor suppressor genes (Table 2). MS‐MLPA was followed by evaluation of several genes using two probes capable of recognizing different Hha1 restriction sites in their promoter regions, several experimental procedures (using MLPA kit) conducted according to manufacturer's instructions, and analysis of signal peak sizes to identify methylation status.

**Table 2:** Information of the genes analyzed by the methylation‐specific multiplex ligation‐dependent probe amplification Kit ME002

Gene	Name	Chromosomal localization
TIMP3	Tissue inhibitor of metalloproteinase 3	22q12.3
APC	Adenomatous polyposis coli	5q21
CDKN2A	Cyclin‐dependent kinase inhibitor 2A	9p21
MLH1	Human mutL homolog 1	3p21.3
ATM	Ataxia telangiectasia mutated	11q22.3
RARB	Retinoic acid receptor	3p24
CDKN2B	Cyclin‐dependent kinase inhibitor 2B	9q21
HIC1	Hypermethylated in cancer 1	17p13.3
CHFR	Checkpoint with forkhead and ring finger domains	12q24.33
BRCA1	Breast cancer 1	17q21
CASP8	Caspase 8	2q33‐q34
CDKN1B	Cyclin‐dependent kinase inhibitor 1B	12p13.1
PTEN	Phosphatase and tensin homolog	10q23.31
BRCA2	Breast cancer 2	13q12
CD44	CD44 molecule	11p13
RASSF1A	Ras‐association domain family member 1	3p21.3
DAPK1	Death‐associated protein kinase 1	9q34.1
VHL	Von Hippel‐Lindau	3p26‐p25
ESR1	Estrogen receptor 1	6q25.1
TP73	Tumor protein p73	1p36
FHIT	Fragile histidine triad	3p14.2
IGSF4	Cell adhesion molecule 1	11q23
CDH13	Cadherin 13	16q24.2
GSTP1	Glutathione S‐transferase p1	11q13

### Quantitative microsatellite analysis

Quantitative microsatellite analysis (QuMA) was performed to examine the microsatellite markers D1S507, D1S463, D1S162, D1S214, D1S2795, and D1S464 on 1p and D19S408, D19S926, and D19S606 on 19q. A process that uses Taqman real‐time PCR, QuMA is based on amplification of microsatellite loci that contain (CA)_n_ repeats, where the repeat is the target for hybridization by the fluorescence‐labeled probe CACACACACACACACACACACACACACAC (Purigo, Taipei, Taiwan). Using different flanking primers that had been designed with Primer Express (ABI, Foster City, CA), the single probe was used to determine the copy number of microsatellite loci distributed throughout the human genome (see Table 3 for a detailed list of primers). A reference pool of six loci of chromosomes (D2S385, D3S1554, D5S643, D8S1800, D12S1699, and D21S1904) was used as a control pool. The pooled standard deviation (SD) for all markers in normal DNA was used to calculate a tolerance interval (TI), as had been described by Nigro et al. 12. When all the loci on the same arm of a chromosome were determined to have been deleted, loss of 1p or 19q was concluded, and copy numbers <1.45 were considered to be losses.

**Table 3:** Summary of primer sequences used for quantitative microsatellite analysis

Microsatellite marker	Forward primer	Reverse primer
D1S507	5′‐GAAAGCCACAAACCCTCTTCAC‐3′	5′‐GGATGGGCTCTAGGGTTTCTG‐3′
D1S463	5′‐GCCTGAAGCAATGAATAACAGTTG‐3′	5′‐CTTTTAAGCCTTTTAGTTAGTCTGAGTTTGT‐3′
D1S162	5′‐ACCTTCGGGTTATCCAACAAACT‐3′	5′‐GGGAAAGCCGCCAACAG‐3′
D1S214	5′‐GCCCGAATGACAAGGTGAGA‐3′	5′‐CATTCTGCATTCCTAAAAGCCAGTA‐3′
D1S2795	5′‐ATGTCTCCTCACTTAGTTGGATTAGACA‐3′	5′‐ACCACAGCCTCAGGCTTCTG‐3′
D1S464	5′‐GATGCATTTCATTTTGGCATAGAA‐3′	5′‐GGCCTAAAAATCTTAAACATAGCATAGC‐3′
D19S408	5′‐CGCAAGCCTGAAGTATGTGCTA‐3′	5′‐GAGAACCAACTCATCTTTATTAAATGCA‐3′
D19S926	5′‐TTAGGCCATGATCCCAGGTTTA‐3′	5′‐CAGTGGCCTTATGCGTGAGTAG‐3′
D19S606	5′‐CCCTCCGTGGGCACTGT‐3′	5′‐AGGTACGAGGCTGTGCCTGTAG‐3′

### MIB‐1 immunohistochemistry

For immunohistochemical staining, a 5‐*μ*m section of the tumor tissue was deparaffinized, rehydrated, and subjected to antigen retrieval (Trilogy, Cell Marque, Hot Springs, AR). After cooling for 20 min at room temperature, the retrieval solution was decanted and the sample washed three times at room temperature using a phosphate‐buffered saline solution. After tissue blocking using a commercial blocking solution (Dual Endogenous Enzyme‐Blocking Reagent, Dakocytomation), the primary antibodies for Ki‐67 (1:100, Dako; MIB‐1) were added, and the specimen was incubated at 4°C overnight. Ki‐67 staining was then performed using the Ventana Autostainer (iVIEW DAB Detection Kit, Ventana Medical Systems, Tuscon, AZ) before all sections were counterstained with hemalum. Two observers reviewed each slide and performed Ki‐67 scoring by determining the percentage of positive nuclei from regions of maximal nuclear staining after counting more than 600 cells at ×400 magnification.

### MS‐MLPA data analysis

The peak sizes of MS‐MLPA were exported to an Excel file for determination of aberrant methylation, which can be identified by the appearance of a signal peak after *Hha*I digestion that had been absent in the normal blood‐derived DNA. Normalization of the peak area of each probe was performed by dividing it by the combined areas of the nearest control probes. The relative peak area of each target probe from the digested sample was compared with that of the undigested sample. For each probe, methylation was scored when the calculated ratio was more than 15%.

### Statistical analysis

SPSS Version 15.0 for Windows was used to perform all statistical analysis and a significance level of *P* < 0.05 considered an indication of statistical significance in the examination of data. For survival analysis, multiple comparisons were made and a Bonferroni‐corrected *P*‐value of 0.002 was viewed as significant. The chi‐square test was used to compare the frequencies of promoter hypermethylation according to clinicopathological parameters in brain tumors; the Fisher's exact test to examine data with lower than expected values; the log‐rank test to analyze the association between the methylation of genes and progression‐free or overall survival, with progression‐free survival calculated from surgery to tumor progression or relapse and overall survival defined as the duration between surgery and death; and Kaplan–Meier survival analysis to determine whether MS‐MLPA could be used to differentiate among patients according to clinical outcome, including according to extent of progression‐free and overall survival. Multivariate survival analysis using Cox's regression model was performed to determine the independent predictors for patient survival. Covariates in this model were selected based on context knowledge and previous reported significant genetic markers. Interaction between significant variables was examined by Cox's regression model adjusting for patient's age, chromosome 19q loss, and Ki67 proliferative index. Correlation between Ki67 proliferative index and methylation status of genes was examined by Student's *t*‐test.

## Results

### Clinical characteristics

Clinical data, including sex, age and pathological diagnosis, were obtained from the medical records (Table 1). They included 27 women (44.3%) and 34 men (55.7%). The mean follow‐up period from surgery was 87.1 months (range, 15–195 months). Adjuvant therapy was not performed in 25 patients (41.0%); 10 (16.4%) received both radiotherapy and chemotherapy, 25 (41.0%) received only radiotherapy and one (1.6%) only chemotherapy. A combination regimen of procarbazine, lomustine, and vincristine was the most commonly used. Fifty‐seven percent of the patients had frontal lobe tumors or both the frontal and other lobes were involved.

### Correlation between genetic alterations and prognosis

**Figure 1:**
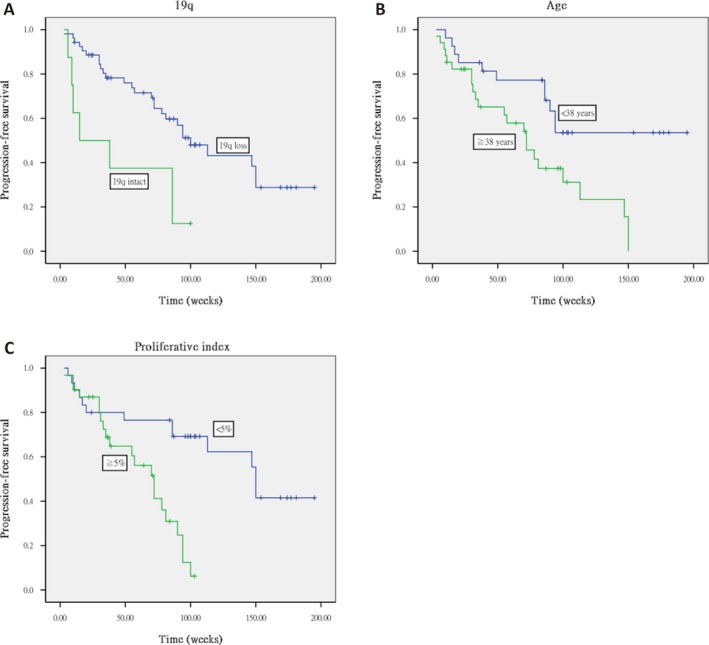
Kaplan–Meier curve survival analysis indicating that progression‐free survival was significantly longer with (A) 19q loss (log rank, *P* = 0.049), (B) age less than 38 years (log rank, *P* = 0.037), and (C) proliferative index of less than 5% (log rank, *P* = 0.003).

**Figure 2:**
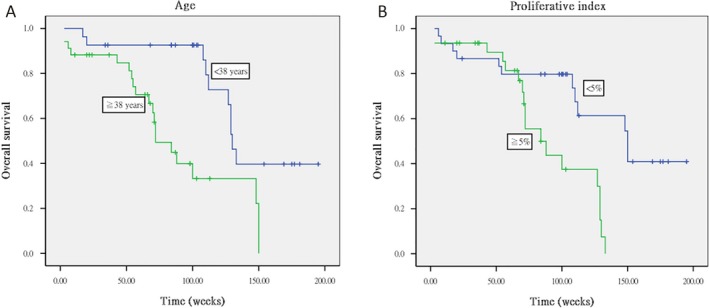
Kaplan–Meier curve survival analysis indicating that overall survival was significantly longer with (A) age less than 38 years (log rank, *P* = 0.007) and (B) proliferative index of less than 5% (log rank, *P* = 0.006).

Copy numbers at six loci on 1p and three loci on 19q were identified in all 61 tumors, while intact 1p and 19q was found on six tumors. The frequencies of deletions in regions 1p and 19q and of 1p/19q codeletion were found to be 70.5%, 88.5%, and 68.9%, respectively. The results of Kaplan–Meier survival curve analysis indicated that progression‐free survival duration was significantly longer in patients with 19q loss (log rank, *P* = 0.049; Fig. 1A) and who were under 38 years of age (log rank, *P* = 0.037; Fig. 1B), and that overall survival was significantly longer in patients who were under 38 years of age (log rank, *P* = 0.007; Fig. 2A).

### Correlation between Ki‐67 proliferative index and prognosis

A Ki‐67 labeling index (LI) of 4.11 ± 5.14, 21.65 ± 10.66, and 7.15 ± 6.86 was found for the grade II oligodendrogliomas, grade III oligodendrogliomas, and grade II oligoastrocytomas, respectively. Based on the MIB‐1 labeling in proliferating cells, a proliferative index of less than 5% is a useful prognostic factor for both progression‐free survival (log rank, *P* = 0.003; Fig. 1C) and overall survival in this study (log rank, *P* = 0.006; Fig. 2B).

### MS‐MLPA profiles

Overall, the most frequently hypermethylated genes identified by MS‐MLPA were RASSF1A, CASP8, and CDKN2A/p16, which were detected in 80.3%, 70.5%, and 52.5% of the cases, respectively (Table 4). As can be observed in Table 4, which shows the frequency of methylation of the genes in oligodendroglial tumors based on clinicopathologic variables, no methylation of CHFR, PTEN, or VHL was detected in the samples tested.

**Table 4:** Frequency of gene methylation in patients with oligodendroglial tumors based on clinicopathologic variables

Gene	Overall methylation (%)	Age, years (%)		Gender (%)		Histology (%)		
		<38 (*n* = 27)	≥38 (*n* = 34)	Male	Female	OD II	OD III	OA II
TIMP3	8.2	7.4	8.8	11.8	3.7	7.7	14.3	6.7
APC	1.6	3.7	0.0	2.9	0.0	2.6	0.0	0.0
CDKN2A	52.5	51.9	52.9	52.9	51.9	53.8	71.4	40.0
MLH1	1.6	0.0	2.9	2.9	0.0	2.6	0.0	0.0
ATM	19.7	22.2	17.6	29.4	7.4	23.1	28.6	6.7
RARB	24.6	22.2	26.5	26.5	22.2	20.5	28.6	33.3
CDKN2B	29.5	40.7	20.6	35.3	22.2	30.8	28.6	26.7
HIC1	14.8	22.2	8.8	23.5	3.7	17.9	28.6	0.0
CHFR	0.0	0.0	0.0	0.0	0.0	0.0	0.0	0.0
BRCA1	1.6	0.0	2.9	2.9	0.0	0.0	14.3	0.0
CASP8	70.5	77.8	64.7	73.5	66.7	76.9	71.4	53.3
CDKN1B	4.9	11.1	0.0	8.8	0.0	7.7	0.0	0.0
PTEN	0.0	0.0	0.0	0.0	0.0	0.0	0.0	0.0
BRCA2	11.5	22.2	2.9	14.7	7.4	12.8	0.0	13.3
CD44	21.3	33.3	11.8	23.5	18.5	25.6	0.0	20.0
RASSF1A	80.3	70.4	88.2	79.4	81.5	84.6	100.0	60.0
DAPK1	4.9	7.4	2.9	8.8	0.0	7.7	0.0	0.0
VHL	0.0	0.0	0.0	0.0	0.0	0.0	0.0	0.0
ESR1	14.8	11.1	17.6	8.8	22.2	12.8	28.6	13.3
TP73	13.1	18.5	8.8	17.6	7.4	15.4	28.6	0.0
FHIT	9.8	11.1	8.8	11.8	7.4	15.4	0.0	0.0
IGSF4	9.8	14.8	5.9	8.8	11.1	12.8	0.0	6.7
CDH13	4.9	7.4	2.9	2.9	7.4	7.7	0.0	0.0
GSTP1	9.8	18.5	2.9	11.8	7.4	10.3	0.0	13.3

OD II, Grade II oligodendroglioma; OD III, Grade III oligodendroglioma; OA II, Grade II oligoastrocytoma.

Ki67 proliferative index between methylated and unmethylated groups of genes was examined by Student's *t*‐test (Table 5). Methylation of RASSF1A was associated with high Ki67 proliferative index (*P* < 0.001).

**Table 5:** Correlation between Ki67 proliferative index and methylation status of genes

Loci	Ki67		*P*‐value
	Methylated	Unmethylated	
TIMP	0.06 ± 0.08	0.09 ± 0.10	0.61
CDKN2A	0.09 ± 0.09	0.08 ± 0.11	0.57
ATM	0.07 ± 0.08	0.09 ± 0.10	0.57
RARB	0.05 ± 0.06	0.10 ± 0.11	0.06
CDKN2B	0.05 ± 0.06	0.10 ± 0.11	0.07
HIC1	0.06 ± 0.06	0.09 ± 0.10	0.46
CASP8	0.08 ± 0.10	0.11 ± 0.09	0.41
CDKN1B	0.00 ± 0.00	0.09 ± 0.10	0.22
BRCA2	0.08 ± 0.07	0.09 ± 0.10	0.91
CD44	0.05 ± 0.07	0.09 ± 0.11	0.31
RASSF1A	0.10 ± 0.10	0.01 ± 0.01	0.00^a^
DAPK1	0.01 ± 0.01	0.08 ± 0.10	0.27
ESR1	0.16 ± 0.18	0.07 ± 0.08	0.36
TP73	0.06 ± 0.09	0.09 ± 0.10	0.56
FHIT	0.06 ± 0.06	0.09 ± 0.10	0.55
IGSF4	0.07 ± 0.07	0.08 ± 0.10	0.86
CDH13	0.07 ± 0.12	0.09 ± 0.10	0.79
GSTP1	0.03 ± 0.07	0.09 ± 0.10	0.29

a Gene APC and MLH1 and BRCA1 have only one methylated sample and could not be compared with independent *t*‐test.

### Correlation between MS‐MLPA profiles and prognosis

**Figure 3:**
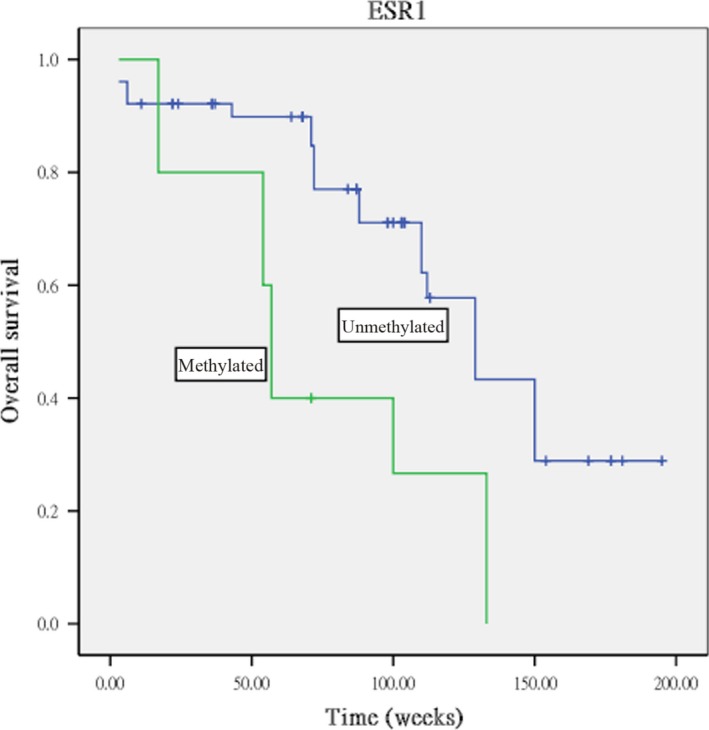
Kaplan–Meier curve survival analysis indicating that tumors methylated for ESR1 showed poor overall survival than those unmethylated for this gene (log rank, *P* = 0.013).

**Figure 4:**
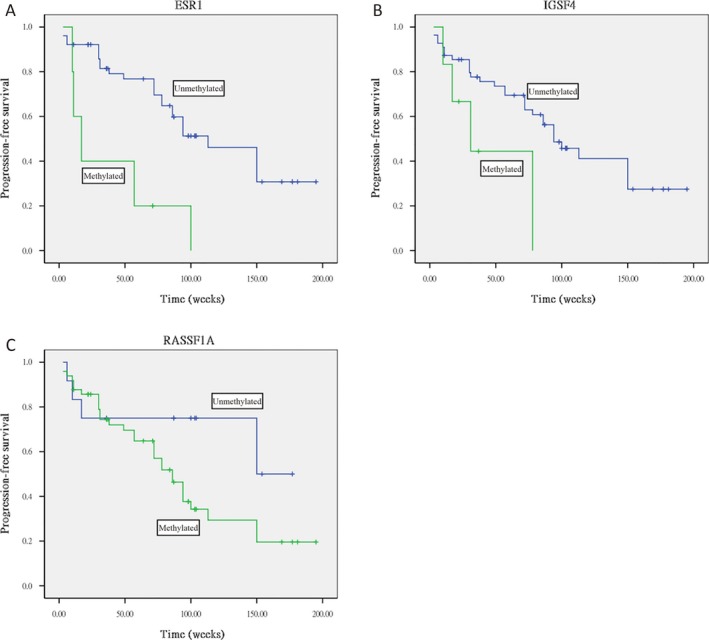
Kaplan–Meier curve survival analysis indicating that (A) tumors methylated for ESR1 showed poor progression‐free survival than those unmethylated for this gene (log rank, *P* = 0.007), (B) tumors methylated for IGSF4 showed poor progression‐free survival than those unmethylated for this gene (log rank, *P* = 0.003), (C) tumors methylated for RASSF1A showed poor progression‐free survival than those unmethylated for this gene (log rank, *P* = 0.039).

The results of Kaplan–Meier survival analysis indicated that overall survival duration was significantly shorter for patients with tumors methylated for the ESR1 gene compared to those with tumors unmethylated for ESR1 (log rank, *P* = 0.013; Fig. 3) and that progression‐free survival duration was significantly shorter for patients with methylation for ESR1 (log rank, *P* = 0.007; Fig. 4A), IGSF4 (log rank, *P* = 0.003; Fig. 4B), and RASSF1A (log rank, *P* = 0.039; Fig. 4C) compared to those with tumors without methylation for ESR1, IGSF4, and RASSF1A.

None of the methylation status of ESR1, IGSF4, and RASSF1A was of prognostic value for survival in a multivariate Cox model when patient's age, chromosome 19q loss, and Ki67 proliferative index were adjusted (Table 6).

**Table 6:** Multivariate analysis of methylation status in relationship to overall survival and progression‐free survival

Loci	Overall survival		Progression‐free survival	
	Hazard ratio (95% CI)^a^	*P*‐value	Hazard ratio (95% CI)^a^	*P*‐value
RASSF1A	3.93 (0.19–2.10)	0.458	0.64 (0.19–2.10)	0.458
IGSF4	6.12 (0.44–34.83)	0.218	0.45 (0.03–6.39)	0.558
ESR1	1.73 (0.55–5.41)	0.347	1.42 (0.37–5.51)	0.612

CI, confidence interval.

a adjusted for patient's age, loss of 19q, and Ki67 proliferative index.

We also examined the two‐way interaction between significant markers by Cox's regression mode and found a significant interaction between RASSF1A and IGSF4, and ESR1 and IGSF4 (Table 7).

**Table 7:** Analysis of two‐way interaction between significant markers

	Overall survival	Progression‐free survival
Interactions	*P*‐value	
19q^a^RSSF1A	0.788	0.378
19q^a^ESR1	0.155	0.584
19q^a^IGSF4	0.090	0.194
ESR1^1^RSSF1A	0.272	0.259
RASSF1A^a^IGSF4	0.378	<0.001
ESR1^a^IGSF4	0.378	<0.001

a adjusted for patient's age and Ki67 proliferative index.

## Discussion

Acquisition of various genetic and epigenetic alterations involving tumor suppressor genes, cell‐cycle regulation genes, and oncogenes may cause extensive changes in the expression of the genes involved in carcinogenesis. Extensive study of the genetic alterations and possible pathways underlying the tumorigenesis of selected brain tumors has indicated that several of these alterations act as early events in tumor development, while others play roles at later or advanced stages. Among the alterations that have been observed, hypermethylation of CpG islands in the promoter regions of tumor suppressor genes has been found to generally lead to the silencing of the respective genes 13. Regarding the gene alterations among specific types of tumors, high incidence of loss of chromosome 1p and 19q and methylation of several genes, such as MGMT, estrogen receptor gene, RB1, TP73, TP14, TP15, and TP16, have been found to be characteristics of oligodendroglial tumors.

In light of these findings, this study evaluated the application of MS‐MLPA in determining the epigenetic profiles of 61 oligodendroglial tumors, including 39 grade II oligodendrogliomas, 7 grade II oligoastrocytomas, and 15 grade III oligodendrogliomas. The ME002 Kit was chosen because it included the analysis of genes playing important roles in cell‐cycle control, transcription regulation, and cell differentiation and proliferation. This kit has been widely used to investigate the methylation status in studies of breast cancer, prostate cancer, and neuroblastoma 14, 15, 16.The results are concordant with previous reports revealing alterations in the methylation profiles of several genes in patients with oligodendroglial tumors, including TIMP3 17, CDKN2A 17, CDKN2B 18, CDKN1B, PTEN 8, RASSF1A 8, DAPK1 17, ESR1 18, TP73 17, and GSTP1 17.

Importantly, this study was the first to identify 14 methylated genes in oligodendroglial tumor patients, namely APC, MLH1, ATM, RARB, HIC1, BRCA1, CASP8, BRCA2, CD44, VHL, FHIT, IGSF4, CDH13, and MLH1. Correlation of clinical outcome with the methylation profiles of these genes, which may help to improve the histopathologic stratification and prognostic prediction in terms of progression‐free survival and overall survival, revealed that individual tumors behaved according to their methylation patterns. These findings highlight the important roles that these genes may play in neoplasm development.

To the best of our knowledge, MLPA has only been used to identify copy number variations in oligodendroglial tumors 19, 20 and, prior to this study, never to analyze the methylation profiles of these tumors. In contrast to methylation‐specific PCR (MSP), which is widely used to detect the methylation status of a single gene or a limited number of genes, MLPA, a method that uses methylation‐sensitive digestion, not only allows screening of several promoters of tumor‐related genes in a sole experiment but also provides semiquantitative data for analysis. The reproducibility and reliability of the results obtained by MLPA, which requires only a small amount of DNA extracted from fresh or formalin‐fixed tumor tissue, have been proven 20. As MLPA was performed in this study using samples obtained by at least partial or total excision instead of tumor biopsy, the risk of failing to analyze the most aggressive part of the tumor, and thus, the risk of making an erroneous diagnosis of a lower‐grade tumor, was relatively low.

The identification of general methylation profiles for oligodendroglial tumors demonstrated the potential impact of these tumor‐related genes on tumor progression. In accordance with previous studies, high methylation rates were found for CDKN2A 17, CDKN2B 18, and RASSF1A 8, suggesting that aberrant methylation of these genes may indicate early change in tumorigenesis of oligodendroglial tumors, regardless of cell type. Both CDKN2A and CDKN2B, tumor suppressor genes encoding p16 (INK4a) and p15 (INK4b), respectively, which are localized to 9p21 and act via the Rb and p53 pathways, have been found to be aberrant to some degree in gliomas 21, 22. More specifically, p16/INK4 has been found to induce G1 cell‐cycle arrest through the Rb pathway, and both p16 and p14ARF have been identified as modulators of chemo‐ and radiosensitivity in gliomas 23. Although 52.5% of the p16 genes in the series examined in this study were found to have been methylated, p16 methylation status was not found to be significantly correlated with the clinical outcome. CASP8, a gene located at 2q33–34 that encodes caspase 8, was found to be methylated in 70.5% of the current series. The most upstream protease of the activation cascade of caspases responsible for the execution‐phase of cell apoptosis, methylation of the CASP8 gene has been reported to be a common epigenetic characteristic in thyroid cancer and breast cancer 24, 25.

The gene found to be most commonly methylated in this study was RASSF1A, which is located at 3p21.3, where it is involved in Ras signaling. As methylation of RASSF1A has been reported in a variety of tumors 24, 26, RASSF1A promoter methylation has been reported to be a useful predictor for clinical outcome in lung cancer, hepatocellular carcinoma, and breast cancer 26, 27, 28. In this study, RASSF1A promoter methylation was found to be associated with shorter duration of progression‐free survival using univariate survival analysis, but this gene was not a prognostic factor in a multivariate Cox model, adjusting for patient's age, chromosome 19q loss, and Ki67 proliferative index.

Aberrant methylation of the ESR1 gene, a ligand‐activated transcription factor located on chromosome 6q24‐q27 that is composed of several domains important for hormone and DNA binding and for transcription activation, has been found to be an independent marker of poorer outcome in laryngeal cancer 29. In this study, methylation of ESR1, which had been previously detected in grade II and grade III oligodendrogliomas but whose clinical correlation with prognosis had not been previously examined 18, was found to be a statistically significant predictor of overall and progression‐free survival using univariate survival analysis. However, this gene was of no prognostic value in a multivariate Cox model, adjusting for patient's age, chromosome 19q loss, and Ki67 proliferative index. IGSF4 gene, a novel immunoglobulin (Ig)‐like intercellular adhesion molecule located on chromosome 11q23, was first characterized as a tumor suppressor of non‐small‐cell lung cancer. Methylation of the gene was found to be associated with poor survival in non‐small‐cell lung carcinoma 30. In accordance with prior research, univariate survival analysis in this study demonstrated that progression‐free survival was found to be significantly shorter for patients with IGSF4 methylation compared to those without IGSF4 methylation. However, IGSF4 was not a prognostic factor in a multivariate Cox model.

Loss of 1p/19q is considered a common early event in tumorigenesis of oligodendroglial tumors. Codeletion of 1p and 19q has been linked to prolonged survival in oligodendroglial tumor patients 31, 32, among those whose tumors have lost the entire arm of chromosomes 1p/19q tend to have a better prognosis than those with tumors having only partial or no loss of these chromosomes 33. In contrast, only 19q deletion and not 1p/19q codeletion was found to be a significant predictor of longer duration of progression‐free survival in the series examined in this study. To demonstrate the proliferative phase of the cell cycle, the MIB monoclonal antibody, a specific marker of proliferation, was used to identify Ki‐67, a nuclear antigen expressed in all phases of the cell cycle except the G0 phase 34. Based on consideration of a Ki‐67 LI of more than 5% as a predictor for shorter duration of progression‐free survival and overall survival, no correlation was found between Ki‐67 LI and 1p/19q status in this study, in accordance with previous studies 8, 35, 36.

The generalizability of the findings of this study may be limited by the four primary limitations faced by this study, namely the relatively small sample examined, the histological heterogeneity of oligodendroglial tumors, various treatments provided, and, most significantly, the limitations inherent in using MLPA. Survival analysis was conducted in the entire group instead of patients in each subgroup of tumors due to the limitation of case numbers. Being based on a single CpG site and analyzing only a small part of the promoter, MLPA cannot provide a complete profile of the methylation status of all CpG sites in a single gene. When using MLPA, a cutoff ratio calculated by dividing the relative peak area of each target probe by that of the undigested sample ranging from 15% to 30% is used 37, 38, 39. In this study, methylation was scored when the ratio was found to be more than 15%.

## Conclusions

The innovative application of MS‐MLPA in this analysis of oligodendroglial tumors allowed for identification of a number of novel and interesting epigenetic alterations, including involving APC, MLH1, ATM, RARB, HIC1, BRCA1, CASP8, BRCA2, CD44, VHL, FHIT, IGSF4, CDH13, and MLH1. Significantly, methylation of ESR1 was found to be significantly associated with shorter duration of progression‐free and overall survival and methylation of IGSF4 and RASSF1A with shorter duration of progression‐free survival using univariate analysis. These findings highlight the importance of these potential biomarkers and their promoter regions on chromosomes and their possible involvement in tumorigenesis, indicating that they play a greater role in the subclassification of certain tumors. These findings also provided hints for designing therapeutic strategies in oligodendroglial tumors, given the reversible nature of epigenetic gene silencing. Larger studies are required to confirm the findings of this study.

## Conflict of Interest

None of the authors have any actual or potential conflicts of interests including any financial, personal, or other relationships with other people or organizations that could inappropriately influence their work.

## References

[1] Issa, J. P. , S. B. Baylin , and J. G. Herman . 1997 DNA methylation changes in hematologic malignancies: biologic and clinical implications. Leukemia 11(Suppl 1):S7–S11. 9130685

[2] Momparler, R. L. , and V. Bovenzi . 2000 DNA methylation and cancer. J. Cell. Physiol. 183:145–154. 10737890 10.1002/(SICI)1097-4652(200005)183:2<145::AID-JCP1>3.0.CO;2-V

[3] Lao, V. V. , and W. M. Grady . 2011 Epigenetics and colorectal cancer. Nat. Rev. Gastroenterol. Hepatol. 8:686–700. 22009203 10.1038/nrgastro.2011.173 PMC3391545

[4] Mitchell, S. M. , J. P. Ross , H. R. Drew , et al. 2014 A panel of genes methylated with high frequency in colorectal cancer. BMC Cancer 14:54. 24485021 10.1186/1471-2407-14-54 PMC3924905

[5] Kaina, B. , and M. Christmann . 2002 DNA repair in resistance to alkylating anticancer drugs. Int. J. Clin. Pharmacol. Ther. 40:354–367. 12467304 10.5414/cpp40354

[6] Gerson, S. L. 2004 MGMT: its role in cancer aetiology and cancer therapeutics. Nat. Rev. Cancer 4:296–307. 15057289 10.1038/nrc1319

[7] Bello, M. J. , C. Aminoso , I. Lopez‐Marin , D. Arjona , P. Gonzalez‐Gomez , M. E. Alonso , et al. 2004 DNA methylation of multiple promoter‐associated CpG islands in meningiomas: relationship with the allelic status at 1p and 22q. Acta Neuropathol. 108:413–421. 15365725 10.1007/s00401-004-0911-6

[8] Kuo, L. T. , K. T. Kuo , M. J. Lee , C. C. Wei , F. Scaravilli , J. C. Tsai , et al. 2009 Correlation among pathology, genetic and epigenetic profiles, and clinical outcome in oligodendroglial tumors. Int. J. Cancer 124:2872–2879. 19330828 10.1002/ijc.24303

[9] Sardi, I. , V. Cetica , M. Massimino , A. M. Buccoliero , L. Giunti , L. Genitori , et al. 2009 Promoter methylation and expression analysis of MGMT in advanced pediatric brain tumors. Oncol. Rep. 22:773–779. 19724855 10.3892/or_00000499

[10] Nygren, A. O. , N. Ameziane , H. M. Duarte , R. N. Vijzelaar , Q. Waisfisz , C. J. Hess , et al. 2005 Methylation‐specific MLPA (MS‐MLPA): simultaneous detection of CpG methylation and copy number changes of up to 40 sequences. Nucleic Acids Res. 33:e128. 16106041 10.1093/nar/gni127 PMC1187824

[11] Lee, J. Y. , C. K. Park , S. H. Park , K. C. Wang , B. K. Cho , and S. K. Kim . 2011 MGMT promoter gene methylation in pediatric glioblastoma: analysis using MS‐MLPA. Childs Nerv. Syst. 27:1877–1883. 21789683 10.1007/s00381-011-1525-7

[12] Nigro, J. M. , M. A. Takahashi , D. G. Ginzinger , M. Law , S. Passe , R. B. Jenkins , et al. 2001 Detection of 1p and 19q loss in oligodendroglioma by quantitative microsatellite analysis, a real‐time quantitative polymerase chain reaction assay. Am. J. Pathol. 158:1253–1262. 11290543 10.1016/S0002-9440(10)64076-X PMC1891922

[13] Cross, S. H. , and A. P. Bird . 1995 CpG islands and genes. Curr. Opin. Genet. Dev. 5:309–314. 7549424 10.1016/0959-437x(95)80044-1

[14] Gumy‐Pause, F. , B. Pardo , M. Khoshbeen‐Boudal , et al. 2012 GSTP1 hypermethylation is associated with reduced protein expression, aggressive disease and prognosis in neuroblastoma. Genes Chromosom. Cancer 51:174–185. 22045684 10.1002/gcc.20941

[15] Moelans, C. B. , A. H. Verschuur‐Maes , and P. J. van Diest . 2011 Frequent promoter hypermethylation of BRCA2, CDH13, MSH6, PAX5, PAX6 and WT1 in ductal carcinoma in situ and invasive breast cancer. J. Pathol. 225:222–231. 21710692 10.1002/path.2930

[16] Schwarzenbach, H. , F. K. Chun , H. Isbarn , et al. 2011 Genomic profiling of cell‐free DNA in blood and bone marrow of prostate cancer patients. J. Cancer Res. Clin. Oncol. 137:811–819. 20683729 10.1007/s00432-010-0941-5 PMC11828105

[17] Alonso, M. E. , M. J. Bello , P. Gonzalez‐Gomez , D. Arjona , J. Lomas , J. M. de Campos , et al. 2003 Aberrant promoter methylation of multiple genes in oligodendrogliomas and ependymomas. Cancer Genet. Cytogenet. 144:134–142. 12850376 10.1016/s0165-4608(02)00928-7

[18] Uhlmann, K. , K. Rohde , C. Zeller , J. Szymas , S. Vogel , K. Marczinek , et al. 2003 Distinct methylation profiles of glioma subtypes. Int. J. Cancer 106:52–59. 12794756 10.1002/ijc.11175

[19] Franco‐Hernandez, C. , V. Martinez‐Glez , M. E. Alonso , J. M. De Campos , A. Isla , J. Vaquero , et al. 2007 Gene dosage and mutational analyses of EGFR in oligodendrogliomas. Int. J. Oncol. 30:209–215. 17143531

[20] Jeuken, J. W. , S. J. Cornelissen , M. Vriezen , M. M. Dekkers , A. Errami , A. Sijben , et al. 2007 MS‐MLPA: an attractive alternative laboratory assay for robust, reliable, and semiquantitative detection of MGMT promoter hypermethylation in gliomas. Lab. Invest. 87:1055–1065. 17700563 10.1038/labinvest.3700664

[21] Schmidt, E. E. , K. Ichimura , G. Reifenberger , and V. P. Collins . 1994 CDKN2 (p16/MTS1) gene deletion or CDK4 amplification occurs in the majority of glioblastomas. Cancer Res. 54:6321–6324. 7987821

[22] Wolter, M. , J. Reifenberger , B. Blaschke , K. Ichimura , E. E. Schmidt , V. P. Collins , et al. 2001 Oligodendroglial tumors frequently demonstrate hypermethylation of the CDKN2A (MTS1, p16INK4a), p14ARF, and CDKN2B (MTS2, p15INK4b) tumor suppressor genes. J. Neuropathol. Exp. Neurol. 60:1170–1180. 11764089 10.1093/jnen/60.12.1170

[23] Simon, M. , D. Voss , T. W. Park‐Simon , R. Mahlberg , and G. Koster . 2006 Role of p16 and p14ARF in radio‐ and chemosensitivity of malignant gliomas. Oncol. Rep. 16:127–132. 16786135

[24] Peters, I. , K. Rehmet , N. Wilke , M. A. Kuczyk , J. Hennenlotter , T. Eilers , et al. 2007 RASSF1A promoter methylation and expression analysis in normal and neoplastic kidney indicates a role in early tumorigenesis. Mol. Cancer. 6:49. 17634119 10.1186/1476-4598-6-49 PMC1939711

[25] Stephen, J. K. , D. Chitale , V. Narra , K. M. Chen , R. Sawhney , and M. J. Worsham . 2011 DNA methylation in thyroid tumorigenesis. Cancers 3:1732–1743. 21738852 10.3390/cancers3021732 PMC3129708

[26] Brock, M. V. , C. M. Hooker , E. Ota‐Machida , Y. Han , M. Guo , S. Ames , et al. 2008 DNA methylation markers and early recurrence in stage I lung cancer. N. Engl. J. Med. 358:1118–1128. 18337602 10.1056/NEJMoa0706550

[27] Buhmeida, A. , A. Merdad , J. Al‐Maghrabi , F. Al‐Thobaiti , M. Ata , A. Bugis , et al. 2011 RASSF1A methylation is predictive of poor prognosis in female breast cancer in a background of overall low methylation frequency. Anticancer Res. 31:2975–2981. 21868547

[28] Huang, Z. H. , Y. Hu , D. Hua , Y. Y. Wu , M. X. Song , and Z. H. Cheng . 2011 Quantitative analysis of multiple methylated genes in plasma for the diagnosis and prognosis of hepatocellular carcinoma. Exp. Mol. Pathol. 91:702–707. 21884695 10.1016/j.yexmp.2011.08.004

[29] Stephen, J. K. , K. M. Chen , V. Shah , S. Havard , A. Kapke , M. Lu , et al. 2010 DNA hypermethylation markers of poor outcome in laryngeal cancer. Clin. Epigenet. 1:61–69. 10.1007/s13148-010-0005-3 PMC3037188 21318053

[30] Kikuchi, S. , D. Yamada , T. Fukami , T. Maruyama , A. Ito , H. Asamura , et al. 2006 Hypermethylation of the TSLC1/IGSF4 promoter is associated with tobacco smoking and a poor prognosis in primary nonsmall cell lung carcinoma. Cancer 106:1751–1758. 16534787 10.1002/cncr.21800

[31] Dehais, C. , F. Laigle‐Donadey , Y. Marie , M. Kujas , J. Lejeune , A. Benouaich‐Amiel , et al. 2006 Prognostic stratification of patients with anaplastic gliomas according to genetic profile. Cancer 107:1891–1897. 16986124 10.1002/cncr.22211

[32] Fallon, K. B. , C. A. Palmer , K. A. Roth , L. B. Nabors , W. Wang , M. Carpenter , et al. 2004 Prognostic value of 1p, 19q, 9p, 10q, and EGFR‐FISH analyses in recurrent oligodendrogliomas. J. Neuropathol. Exp. Neurol. 63:314–322. 15099021 10.1093/jnen/63.4.314

[33] McLendon, R. E. , J. E. II Herndon , B. West , D. Reardon , R. Wiltshire , B. K. Rasheed , et al. 2005 Survival analysis of presumptive prognostic markers among oligodendrogliomas. Cancer 104:1693–1699. 16116609 10.1002/cncr.21362

[34] Darling, J. L. 1991 Ki‐67 monoclonal antibody. Br. J. Neurosurg. 5:438–441. 1664731

[35] Coons, S. W. , P. C. Johnson , and D. K. Pearl . 1997 The prognostic significance of Ki‐67 labeling indices for oligodendrogliomas. Neurosurgery 41:878–884; discussion 884‐875. 9316050 10.1097/00006123-199710000-00021

[36] Heegaard, S. , H. M. Sommer , H. Broholm , and O. Broendstrup . 1995 Proliferating cell nuclear antigen and Ki‐67 immunohistochemistry of oligodendrogliomas with special reference to prognosis. Cancer 76:1809–1813. 8625052 10.1002/1097-0142(19951115)76:10<1809::aid-cncr2820761020>3.0.co;2-i

[37] Bol, G. M. , K. P. Suijkerbuijk , J. Bart , M. Vooijs , E. van der Wall , and P. J. van Diest . 2010 Methylation profiles of hereditary and sporadic ovarian cancer. Histopathology 57:363–370. 20840667 10.1111/j.1365-2559.2010.03642.x

[38] Castro, M. , L. Grau , P. Puerta , L. Gimenez , J. Venditti , S. Quadrelli , et al. 2010 Multiplexed methylation profiles of tumor suppressor genes and clinical outcome in lung cancer. J. Transl. Med. 8:86. 20849603 10.1186/1479-5876-8-86 PMC2955578

[39] Livide, G. , M. C. Epistolato , M. Amenduni , V. Disciglio , A. Marozza , M. A. Mencarelli , et al. 2012 Epigenetic and copy number variation analysis in retinoblastoma by MS‐MLPA. Pathol. Oncol. Res. 18:703–712. 22278416 10.1007/s12253-012-9498-8

